# Genetic Diversity and Demographic History of the Shaggy Soft-Haired Mouse *Abrothrix hirta* (Cricetidae; Abrotrichini)

**DOI:** 10.3389/fgene.2021.642504

**Published:** 2021-03-24

**Authors:** Lourdes Valdez, Guillermo D’Elía

**Affiliations:** ^1^Instituto de Ciencias Ambientales y Evolutivas, Facultad de Ciencias, Universidad Austral de Chile, Valdivia, Chile; ^2^Colección de Mamíferos, Facultad de Ciencias, Universidad Austral de Chile, Valdivia, Chile

**Keywords:** Patagonia, Chile, Argentina, population geomics, historical demography, Pleistocene, conservation, *Supramyomorpha*

## Abstract

Genetic information on species can inform decision making regarding conservation of biodiversity since the response of organisms to changing environments depend, in part, on their genetic makeup. Territories of central-southern Chile and Argentina have undergone a varying degree of impact during the Quaternary, where the response of local fauna and flora was rather species-specific. Here, we focus on the sigmodontine rodent *Abrothrix hirta*, distributed from 35° S in Chile and Argentina to northern Tierra del Fuego. Based on 119,226 transcriptome-derived SNP loci from 46 individuals of *A. hirta*, we described the geographic distribution of the genetic diversity of this species using a maximum likelihood tree, principal component and admixture analyses. We also addressed the demographic history of the main intraspecific lineages of *A. hirta* using GADMA. We found that *A. hirta* exhibited four allopatric intraspecific lineages. Three main genetic groups were identified by a Principal Component Analysis and by Ancestry analysis. The demographic history of *A. hirta* was characterized by recent population stability for populations at the northernmost part of the range, while southern populations experienced a recent population expansion.

## Introduction

Decision making aimed to conserve biodiversity requires information on several aspects of the biological system to be protected. In particular, genetic information can be key when conservation efforts are made to ensure the survival through time of natural populations and the processes in which they are involved (Supple and Shapiro, 2018). The response of individual organisms to changing environmental conditions can be challenging, and their acclimation and adaptive potential are conditioned by their genetic makeup (Fitzpatrick and Edelsparre, 2018). Given the uncertainty of future environmental conditions, as well as the lack of clear knowledge regarding the link between genes and phenotypes, it might be a safe bet to direct conservation efforts to ensure the preservation, when detected, of distinct intraspecific lineages (Moritz, 2002), particularly in a geographic area where climatic fluctuations modeled current genetic features of the local fauna. The concept of Evolutionary Significant Units (ESU) and Management Units (MU) were early advanced with the aim of conserving the evolutionary heritage and current genetic structure of populations, respectively (Moritz, 1994) (see also Ryder, 1986; Casacci et al., 2014). In fact, one of the main levels of Biodiversity is the genetic level, which emphasizes the need to preserve intraspecific lineages (Gaston and Spicer, 2004).

The biota of southern South America (south of 38°S), roughly corresponding to Patagonia, has been subjected to marked climate changes, glaciations, volcanism, tectonics, and seashore shifts over the past thousands of years (Heusser, 1984; Rabassa et al., 1989; McCulloch et al., 2000). Those historical events have driven diverse processes in the local biota, including population fragmentation and genetic divergence, secondary contact, and range shift. These processes left recognizable genetic footprints, whose characterization is the basis to infer the processes. Since the footprints get erased with time and with the occurrence of subsequent events, such inferences have limitations (Avise, 2009). The body of literature accumulated on the phylogeography of the biota from southern South America, including plants (e.g., Premoli et al., 2010; Achimón et al., 2018; Biersma et al., 2018; Nicola et al., 2019), reptiles (e.g., Victoriano et al., 2008; Femenias et al., 2020), amphibians (e.g., Nuñez et al., 2011), fishes (e.g., Ruzzante et al., 2006; Zemlak et al., 2008; Ruzzante et al., 2020), birds (Norambuena et al., 2018), and mammals (e.g., Lessa et al., 2010; Palma et al., 2012), reveals that the responses to Quaternary dynamics were varied and mostly species-specific (e.g., Lessa et al., 2010; Sersic et al., 2011). Two general patterns were described by Lessa et al. (2010) for Patagonian sigmodontine rodents. On the one hand, species would have persisted in Pleistocene refugia outside Patagonia and would have colonized southern areas from there when climatic conditions became favorable after the Last Glacial Maximum (LGM; ca. 20,000 to 10,000 ya). On the other hand, species would have persisted in one or more Patagonian refugia, whose location would vary according to the species, but may include the Valdivian Refugium at coastal and pre-Andean areas of Chile (from ca. 39°S to ca. 43°S), which has been indicated as an area where animal and plant species persisted during the last glaciation (e.g., see Valdez and D’Elía, 2018).

*Abrothrix hirta* is a sigmodontine rodent that was revalidated as a distinct species when it was removed from the synonymy of *Abrothrix longipilis* (Teta and Pardiñas, 2014; Teta et al., 2017). The geographic distribution of *A. hirta* is wide. Its northern limit in Chile is not clear, but it spans from ca 35° S at both sides of the Andes to northern Tierra del Fuego and from the Pacific coast to pre-Andean areas up to 2,000 masl, including some lowland areas of the Patagonia. Across this large distribution, the species inhabits a wide range of vegetal formations, including sclerophyllous forests in the north, pre-Andean shrublands, temperate rainy Valdivian and Magellan Forests, and the contrastingly arid Patagonian steppes east of the Andes. Most of the knowledge on the natural history and ecology of *A. hirta* was described referring to it as *A. longipilis.* However, based on the study areas it is possible, in most cases, to reassign the information to *A. hirta* (e.g., Patterson et al., 1990; Kelt et al., 1994). More recent studies provided information on *A. hirta* regarding cranial morphology (Canto et al., 2017), renal transcriptome (Valdez et al., 2015), and digestive morphology (Naya et al., 2014). Cranial variation is not geographically structured (Teta and Pardiñas, 2014), while variation on digestive morphology is associated with habitat type (i.e., forest vs. steppe; Naya et al., 2014). The phylogeographic pattern of *A. hirta* was addressed in two separate contributions. Palma et al. (2010) examined Chilean populations, where six intraspecific lineages were depicted. The same year, Lessa et al. (2010) studied Argentinean and Chilean specimens of *A. hirta*, identifying two allopatric clades distributed in northern and southern Patagonia, respectively. Considering both studies, there are no association of clades with the main habitat types, namely forest and steppe; lineages rather replace each other latidudinally. In addition, both Patagonian lineages exhibited signals of demographic expansion, which would have occurred before the LGM (Lessa et al., 2010). In any case, both studies, as well as most of those focused on other Patagonian rodents cited above, were based on a single mitochondrial gene. The single-gene approach is limited in the sense that they do not allow distinguishing between the effect of neutral demographic processes from that of natural selection (Avise, 2009). Considering that natural selection operates on individual or on a few genes at the time, while demographic processes affect the whole genome, widening the sampling to a genomic scale would inform on this issue. This is one of the reasons why it is necessary to transcend genetic studies based on one or a few loci to those at genomic scale (e.g., Lessa et al., 2014). In this line, here we use a wide panel of transcriptomic-derived single nucleotide polymorphisms (SNPs) to study the genetic diversity and demographic history of *Abrothrix hirta*. We also aimed to assess the intraspecific genetic structure of the species, and to test the hypotheses raised by Lessa et al. (2010), i.e., the existence of two Patagonian genetic lineages that underwent expansion prior to the LGM.

## Materials and Methods

### Specimen Sampling and Data Collection

**FIGURE 1:**
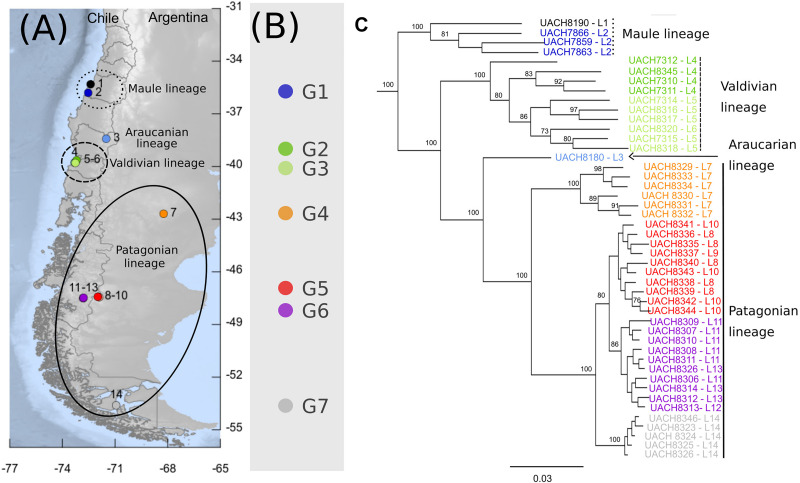
Phylogeographic pattern of *Abrothrix hirta***. (A)** Map of collection localities of *Abrothrix hirta* included in this study. Collection localities are color-coded as in Figure 3. Circles and ellipses indicate the main lineages shown in panel **(C)**. **(B)** Groups of localities and color-coded localities within each group: G1 = 2, G2 = 4; G3 = 5-6, G4 = 7, G5 = 8-10, G6 = 11-13, G7 = 14. Localities included in each group (G1–G7). Details on each locality are provided in Table 1. **(C)** Maximum likelihood tree for samples of *A. hirta.* Node labels correspond to Bootstrap support values. Terminals are color-coded as in panels **(A)** and **(B)**. Lineages are labeled as described in the text.

The geographic sampling covers most of the distribution of the species, based on 46 individuals of *Abrothrix hirta* from 14 localities (Figure 1A and Table 1) present at sclerophyllous shrublands, Valdivian and Magellanic Forests, and Patagonian steppe. Data of 16 specimens from four localities were taken from Valdez et al. (2015), while data on the other 30 individuals from other 10 localities were newly gathered in this study (Table 1). Several of these newly sequenced specimens were already housed at scientific collections. Part of the Chilean specimens were newly collected in this study using Sherman-like traps baited with oatmeal. The taxonomic identity of captured individuals was first determined in the field based on the external morphology; this was later corroborated in the laboratory with sequences of the mitochondrial gene *Cytochrome-b* as in D’Elía et al. (2008). Immediately after euthanasia, the right kidney was removed and conserved in RNAlater stabilization solution (Invitrogen). Specimen vouchers and tissue samples were deposited at the Colección de Mamíferos, Universidad Austral de Chile, Valdivia, Chile. All procedures involving live animals were conducted following guidelines of the American Society of Mammalogists (Sikes and Animal Care and Use Committee of the American Society of Mammalogists, 2016) and were approved by the Uso de Animales en la Investigación committee of the Universidad Austral de Chile (permits 04/11 and 296/2017); the Servicio Agrícola y Ganadero authorized the collection of individuals (permits 1231/2017, 5611/2013, 2164 and 5165).

**TABLE 1:** Specimens of *Abrothrix hirta* included in this study.

**#**	**Locality**	**Specimen ID**	**Group for Fst-based analyses**
1	Chile, Región del Maule, Constitución, Quivolgo; -35.315; -72.395.	UACH 8190	Not included
2*	Chile, Región del Maule, Cauquenes, Pelluhue; -35.80165; -72.5301.	UACH 7859 UACH 7863 UACH 7866	G1
3	Chile, Región de la Araucanía, Malleco, Cordillera Las Raíces, Malalcahuello; -38.419535; -71.505124.	UACH 8180	Not included
4*	Chile, Región de Los Ríos, Valdivia, San José de la Mariquina, Fundo San Martín; -39.651; -73.19.	UACH 7310 UACH 7311 UACH 7312 UACH 7313	G2
5*	Chile, Región de los Ríos, Valdivia, Valdivia, Chabelita; -39.776967; -73.304783.	UACH 7314 UACH 7315, UACH 8316, UACH 8317, UACH 8318	G3
6	Chile, Región de los Ríos, Valdivia, Isla Teja, Campus Universidad Austral de Chile; -39.795101; -73.260741.	UACH 8320	G3
7*	Argentina, Provincia de Chubut, Telsen, Gan Gan, Establecimiento La Maroma; -42.694353; -68.233911.	UACH 8329, UACH 8330, UACH 8331, UACH 8332, UACH 8333, UACH 8334	G4
8	Argentina, Santa Cruz, Río Chico, Cajón del Río Oro, 3 km al O por camino del puente Río Oro; -47.421050; -71.958233.	UACH 8335, UACH 8336, UACH 8338, UACH 8339, UACH 8340	G5
9*	Argentina, Santa Cruz, Río Chico, La península, 2 km al SSO por camino de Tío Camping (EA, La Península); -47.447417; -71.903483.	UACH 8337	G5
10	Argentina, Santa Cruz, Río Chico, Valle del Río Oro, 5 km por camino del puente Río Oro; -47.422150; -71.981567.	UACH 8341, UACH 8342, UACH 8343, UACH 8344	G5
11*	Chile, Región de Aysén, Sector Barrancoso 40 km al sur de Cochrane por ruta 7 (carretera austral); -47.496717; -72.808617.	UACH 8306, UACH 8307, UACH 8308, UACH 8309, UACH 8310, UACH 8311, UACH 8312	G6
12	Chile, Región de Aysén, Sector Barrancoso 41,5 km al sur de Cochrane por ruta 7 (carretera austral); -47.500267; -72.821050.	UACH 8313	G6
13	Chile, Región de Aysén, Sector Barrancoso, 35 km al sur de Cochrane por ruta 7 (carretera austral); -47.477717; -72.794483.	UACH 8314, UACH 8315	G6
14*	Chile, Región de Magallanes, Punta Arenas, Puerto del Hambre; -53.606; -70.937.	UACH 8323, UACH 8324, UACH 8325, UACH 8326, UACH 8346	G7

*The acronym UACH corresponds to the Colección de Mamíferos, Universidad Austral de Chile, where specimens and tissue samples were deposited. Localities included in the IBD analysis are indicated with asterisks.*

### Transcriptome Sequencing and SNP Calling

Total RNA extraction, messenger-RNA purification, and complementary-DNA construction were conducted using RNeasy mini kit (Quiagen), PolyATract mRNA Isolation System II, and TruSeq RNA Sample Preparation Kit, respectively, following Valdez et al. (2015). Libraries constructed for each specimen were subjected to high-throughput sequencing following Illumina HiSeq2000 protocol (Illumina Inc.) to obtain paired-end reads with a length of 101 bp.

Transcriptome data from each of the 46 samples were analyzed individually. Raw reads were trimmed to remove Illumina adapters and low quality reads using Trimgalore tool^1^. Thresholds for minimum quality and read length were set to 20 phred and 25 bp, respectively. Reads derived from ribosomal RNA (rRNA) were excluded from the dataset; these reads were identified mapping trimmed reads using Bowtie version 2.2.6 (Langmead and Salzberg, 2012) against a reference containing rRNA sequences from Rodentia species available at GenBank^2^. Filtered read datasets were assembled *de novo* using Trinity version 2.8.6 (Grabherr et al., 2011). Assembled contigs were annotated using the Blastx alignment tool (Camacho et al., 2009); a dataset containing amino acid sequences of *Mus musculus* from OMA^3^ was used as a reference for gene annotation. After annotating individual assemblies, the best contig was selected for each gene; i.e., the longest contig that spans at least 80% of the amino acid sequence of the corresponding reference protein. As in Giorello et al. (2014), custom scripts were used in this step. The set of such best contigs were used as a reference transcriptome for Single Nucleotide Polymorphism (SNP) calling.

For each sample, SNPs were identified using GATK v.4.1.4.0 (McKenna et al., 2010), following the analytical workflow recommended for RNA-seq data. First, the STAR’s two-pass mode (Dobin et al., 2013) was applied to individual read files against the reference transcriptome. This accounts for possible splice junctions in the dataset. Following this step, GATK’s SplitNCigarReads was used to split reads into exon segments and hard-clip any sequences overhanging into the intronic regions. Duplicated reads that are likely to have been generated due to artifactual processes (such as PCR duplicates) were removed using MarkDuplicates tool from Picard (Wouters et al., 2002). GATK’s HaplotypeCaller was used to generate individual vcf files for each sample. All 46 vcf files were then merged into one file using BCFtools (Danecek and McCarthy, 2017). Only SNP loci present in all 46 individuals were included in downstream analyses. Loci departing from Hardy-Weinberg equilibrium, multiallelic loci, and having a minor allele lower than 0.01 were also filtered out. A total of 119,226 SNP loci were kept after filtering.

### Datasets Used in the Analyses

Three datasets were constructed; all of them containing biallelic SNP loci that were in Hardy-Weinberg equilibrium, present in frequencies higher than 0.01. Filtering of the data was conducted using bcftools^4^. The first one (hereafter dataset 1) contains 119,226 SNP loci from all 46 sampled individuals of *Abrothrix hirta* (Figure 1A and Table 1). The second dataset (hereafter dataset 2) includes 119,226 SNPs loci from 44 individuals from 12 localities; it excludes samples from localities 1 and 3 (Figure 1A and Table 1) because only a single specimen was included from these localities. Dataset 2 was used for those analyses that require multiple sampling per locality (see below). To improve statistical power, geographically close localities were merged into 7 groups in the dataset 2 (G1–G7; see Figure 1B and Table 1). G1 includes samples from the Chilean region of Maule; G2 and G3 contain samples from Los Rios Region. Groups G4 and G5 are distributed in the Argentinean provinces of Chubut and Santa Cruz, respectively (these are all the samples from open arid areas east of the Andes); G6 includes samples from the Chilean Region of Aysén. Finally, G7 is form by samples from the Magallanes Region of Chile (Figure 1C and Table 1). The third dataset (hereafter dataset 3) contains 220,080 SNPs loci from the 46 individuals of *A. hirta* (Table 1), and a one individual of the closely related species *Abrothrix longipilis* (UACH 8097), *A. manni* (UACH 7876), and *A. sanborni* (UACH 8322); the last three were used to form the outgroup, since they are the most closely related species to *A. hirta* (Cañón et al., 2014; Teta et al., 2017).

### Genetic Diversity

Nucleotide diversity (Pi) was calculated using dataset 2, where a single value was obtained for each locality group (G1–G7). To calculate Pi, the average number of nucleotide differences between two randomly chosen samples from within groups of populations was estimated using PopGenome (Pfeifer et al., 2014); this number was then divided by the total number of SNP loci in the dataset. The observed heterozygosity (oHe) for each site and the mean oHe for each population group (G1–G7; Table 1) were calculated using the R package pegas (Paradis, 2010). The fixation index Fst was calculated among pairs of groups using PopGenome.

### Intraspecific Lineages

In order to detect and describe the main intraspecific lineages of *Abrothrix hirta*, a maximum likelihood tree was inferred using dataset 3 and IQ-TREE (Nguyen et al., 2015). The best-fit substitution model for dataset 3 was TM + F + I + G4 + ASC, which was selected based on the Bayesian Information Criterion and implemented in the ML tree reconstruction. Branch support was estimated using 1000 replicates and ultrafast bootstrap (Hoang et al., 2018). An additional step was run to further optimize ultrafast bootstrap trees by nearest-neighbor interchange to account for model violations.

### Genetic Structure

Two approaches were implemented to explore the genetic structure of *A. hirta*. The first one, using dataset 1, consisted in a principal component analysis (PCA) conducted with Plink 1.9 (Purcell et al., 2007). The top 20 principal components of the variance-standardized relationship matrix were extracted. The eigenvectors with the first three principal components were plotted. The second approach consisted in a maximum likelihood estimation of individual ancestry, which was conducted using the R package LEA (Frichot and François, 2015). Individual admixture coefficients were estimated from the genotypic matrix assuming K ancestral populations. Ten independent runs were performed with K = 1 to K = 10 and calculating individual ancestry proportion and a cross-entropy criterion. The run yielding the value of cross-entropy criterion has the number of ancestral population (K) that best explains the genetic dataset (Alexander and Lange, 2011).

In order to test for a pattern of isolation by distance, Mantel statistics were calculated based on Pearson’s product-moment correlation using Fst estimates between pairs of groups (G1–G7), calculated using dataset 2, and geographic distances among one representative of each group of localities and estimated in decimal degrees using the package sp (Pebesma and Bivand, 2005).

### Demographic Modeling

Here the units of analysis were the four uncovered main lineages (see below). One of these (the so-called Araucarian lineage; see results, Figure 1C) was excluded because it is composed by a single sample (UACH 8180) precluding its consideration in this analysis. The site frequency spectrum (sfs) was estimated using easySFS^5^. A projection of 8, 8, 8 was used to generate a joint sfs file, which was the input file for the demographic analysis using GADMA (Noskova et al., 2020). The method of ordinary differential equations was applied using moments software (Jouganous et al., 2017) implemented in GADMA. Running parameters where Theta0: 0.37976, which was calculated as 4^∗^mu^∗^L, as in Gutenkunst et al. (2009); time for generation: 1.0, number of repeats: 30; the remaining parameters were set as default. Several combinations of initial and final structure were previously run and the combination of Initial structure: 1, 1, 1, Final structure: 2, 2, 2; showed the best likelihood value. Demographic models with this structure were evaluated calculating parameters of (a) time of population divergence, (b) effective population size, and (c) migration, for three *a priori* defined populations, namely, the Maule lineage, the Valdivian Lineage, and the Patagonian lineage. Heuristic searches by means of the genetic algorithm (GA) implemented by GADMA was used to find the best-fit parameter values in order to automatically infer the best demographic model from the joint sfs data. Composite Likelihood Akaike Information Criterion (CLAIC) was used to choose the best model considering the three populations in one.

## Results

### Genetic Diversity of *Abrothrix hirta*

**FIGURE 2:**
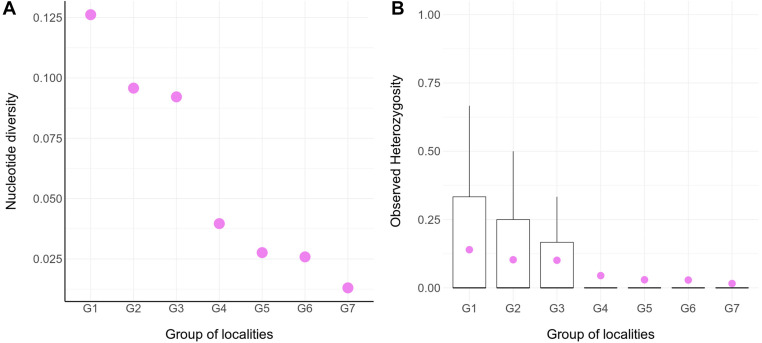
Molecular diversity for seven groups of populations of *Abrothrix hirta*. **(A)** Molecular diversity index (Pi) calculated for seven groups of localities (G1- G7; Figure 1; Table 1). The values for this graph are given in Supplementary Table 1. **(B)** Observed heterozygosity across individuals within groups. The data for this graph is given in Supplementary Table 2.

The genetic diversity within locality groups (G1–G7; Figure 1B and Table 1) was higher at northern localities and gradually decreased toward the south (Figure 2); this pattern was detected with both Pi and oHet estimates. Pi values ranged between 0.01 (for G7) and 0.12 (for G1; Figure 2A and Supplementary Table 1); oHet for individual loci ranged from 0 to 1, but the frequency of each value varied (Figure 2B) and the mean oHet decreased at higher latitudes (Supplementary Table 2).

Pairwise estimates of Fst between group pairs ranged from 0.04 for the comparison of G2 and G3, which are relatively close to each other in the Valdivian forests of the Los Ríos Region, to 0.28 for the comparison between the distantly located groups G4 in the Patagonian steppes of Gan Gan, Chubut, Argentina and G7 in the Magellanic forests of Puerto del Hambre, Magallanes, Chile (Supplementary Table 3).

### Intraspecific Lineages in *Abrothrix hirta*

The ML tree of 46 individuals of *Abrothrix hirta* (Figure 1C) strongly supports the monophyly of the species (BS = 100). Four main intraspecific lineages were recovered, which are geographically segregated and latitudinally ordered. Specimens from the northernmost localities in the Region del Maule (localities 1 and 2: G1; Figure 1 and Table 1) form a strongly supported clade (BS = 100), referred hereafter as the Maule lineage. Specimens from Región de Los Ríos (localities 4, 5, and 6: G2 and G3; Figure 1A and Table 1) form a strongly supported clade (BS = 100), referred here as the Valdivian lineage. A single sample from Malalcahuello (locality 3, Figure 1 and Table 1) comprises the Araucarian lineage. Finally, unlike the previously mentioned lineages, the last main lineage, referred here as the Patagonian lineage, has a broad geographic distribution; it includes individuals from locality 7 (G4) in Gan Gan, Argentina, southwards to localities 8-10 (G5) in Santa Cruz, Argentina, 11-13 (G6), Aysen, Chile and 14 (G7), Puerto del Hambre, Magallanes Region, Chile. Interestingly, specimens of each of these four locality groups form monophyletic groups. Relationships among main lineages are as follows (Maule (Valdivian (Araucarian, Patagonian))); all relationships are highly supported (BS = 100). Branch lengths in the ML tree showed that the Maule and Valdivian lineages contain samples that are more divergent among them in comparison to those in the Patagonian lineage (Figure 1A).

### Genetic Structure of *Abrothrix hirta*

**FIGURE 3:**
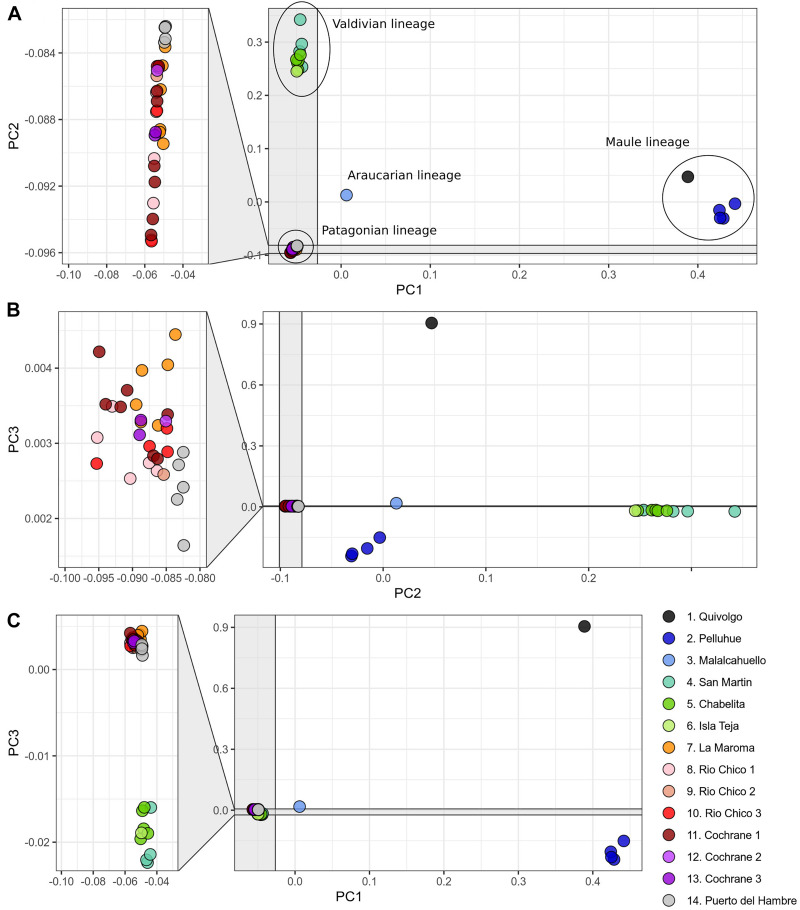
Principal component analysis based on 119,226 SNP loci of 46 specimens of *Abrothrix hirta.* Each specimen was colored according to the locality at which it was collected as indicated in Figure 1 (details in Table 1). Lineages found in the phylogenetic analysis (Figure 1) are indicated with the ellipses. **(A)** PC1 vs PC2; **(B)** PC2 vs PC3; **(C)** PC1 vs PC3.

The PCA conducted with 119,226 SNP loci from all 46 individuals indicates that the species genetic variation is geographically structured (Figure 3 and Supplementary Tables 4, 5). The first three principal components explained 32.41% of the observed variance (Supplementary Table 4). In the two-dimensional space composed by PC1 and PC2 (Figure 3A), four groups of specimens are well segregated in the space; these groups are concordant with the main clades obtained in the ML tree (Figure 1C). The first PCA group, which is the one most segregated from the other groups along PC1, contains samples from localities 1 and 2, corresponding to Maule lineage. This group has a higher dispersion along PC3 than along PC1 and PC2 (Figure 3C). At low PC1 and high PC2 values (Figure 3A) is found a second group of samples from localities 3-6, corresponding to the Valdivian lineage. Samples from localities 7-14, corresponding to the Patagonian lineage, are closely grouped at low PC1 and PC2 values. The single sample from the Araucarian lineage (locality 3) approaches those of the Patagonian lineage (Figure 3A), but in PC2 vs. PC3, it appears closer to samples of the Maule lineage. The segregation of Valdivian and Patagonian samples is low along PC3 (Figure 3C). It is noteworthy that samples of the Patagonian lineage are closely grouped along the first three principal components.

A pattern of IBD was detected by the Mantel test; there is a positive and significant correlation (*r* = 0.6232; *p*-value = 0.007996) of Fst between pairs of groups (G1-G7) and their geographic distances (Supplementary Tables 3, 6, respectively).

**FIGURE 4:**
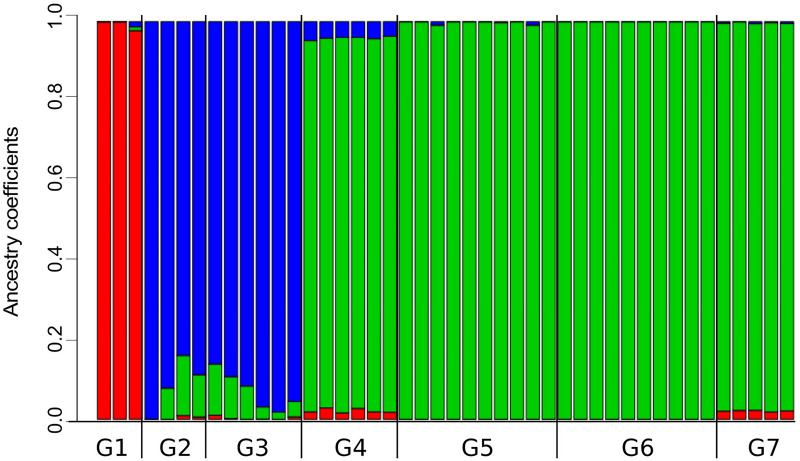
Proportions of ancestry for specimens of *Abrothrix hirta* from seven groups of localities into three genetic clusters. The three genetic clusters (K = 3; see Supplementary Figure 1) are differently colored (red, blue, and green). The black bar below indicates the limits of each of the seven population groups (Figure 1). Samples and localities included in each group are specified in Table 1.

The analysis of population structure with LEA found that the best number of ancestral population (i.e., genetic clusters) in the dataset is K = 3, which yielded the lowest value of cross-entropy (Supplementary Figure 1). A scheme of K = 4 was the second best. Note that the single individual representing the Araucarian lineage was not included in this frequency-based analysis. Individuals from G1 share high coefficients of ancestry corresponding to cluster 1 (Figure 4); moreover, cluster 1 is almost restricted to the Maule lineage (G1), except for the low proportions of this cluster present in individuals from G2, G3, G4, and G7. Individuals from G2 and G3 present high proportion of ancestry corresponding to cluster 2 and small proportions of clusters 1 and 3 (i.e., small sections of red and green in otherwise blue sections; Figure 4). Despite this, cluster 2 roughly approximates the Valdivian lineage (Figure 1C). Similarly, cluster 3, which is present in high proportions of ancestry in individuals from G2 to G7, roughly corresponds to the Patagonian lineage (Figure 1C). In the scheme of K = 4, the single difference consists in a split of cluster 1 into two. The index of fixation, Fst, among the three clusters were very similar; Fst of cluster 1 (red in Figure 4) vs. cluster 2 (blue in Figure 4) = 0.271; Fst of cluster 1 vs cluster 3 (green in Figure 4) = 0.277; and Fst of cluster 2 vs. Cluster 3 = 0.270.

### Historical Demography of *Abrothrix hirta*

**FIGURE 5:**
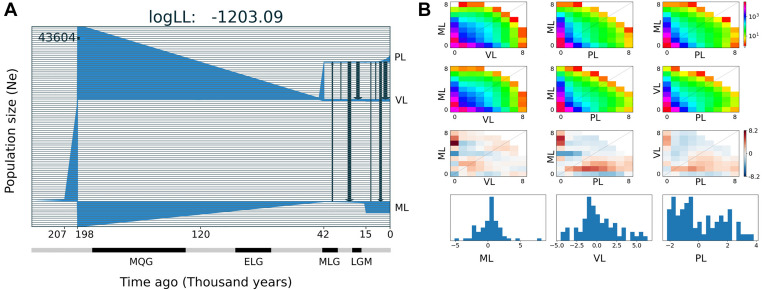
Demographic history for three main lineages of *Abrothrix hirta.* **(A)** Demographic model of the three lineages of *A. hirta*: ML, Maule lineage, VL, Valdivian lineage, and PL, Patagonian lineage. The number at the top-right scales the *y*-axis that corresponds to effective population size. In the x-axis, time is indicated in thousands of years before present. Main glaciation events are indicated in the time axis; MQG, Middle Quaternary Glaciation, ELG, Early Late Quaternary Glaciation; MLG, Middle Late Quaternary Glaciation, LGM, Late Glacial Maximum. **(B)** Allele frequency spectrum distribution of sfs files (first line); theoretical model sfs (second line); residuals (third line); model adjustment for each lineage (fourth line).

The best demographic scenario estimated with GADMA for the three main lineages of *A. hirta* for which more than one specimen was included in the analysis, involves periods of population size stability and change, as well as events of gene flow between lineage pairs; it is described below in six-time intervals (separated by tick marks in the *x*-axis in Figure 5). The ancestral population of *A. hirta* was stable, with an initial Ne of 43,604 individuals until 207 Kya when it underwent a linear growth up in size until 198 Kya when this ancestral population split into two lineages. On the one hand arose the Maule lineage (Figure 5) and on the other hand the ancestral population of the current Valdivian and Patagonian lineages. The Maule lineage experienced a steady reduction of size until 42 Kya, to persist at minimal values until it underwent a sudden population growth 15 Kya ago, since when it maintained a stable size until the present. The ancestral population of Valdivian and Patagonian lineages also experienced a marked and continuous reduction in size until 42 Kya when it split into current Valdivian and Patagonian lineages. Since its origin, the Ne of the Valdivian lineage remained stable, while the Patagonian lineage experienced exponential population growth during the last ca. 7,500 years. Additionally, during the last ca. 40 Kya, events of bidirectional and asymmetric gene flow between Maule and Patagonian lineages, as well as between Valdivian and Patagonian lineages have taken place (black arrows in Figure 5).

## Discussion

*Abrothrix hirta* is one of the most abundant rodents in southern South America (Patterson et al., 2015). However, the northern limit of its distribution remains unclear. As a result of our field work, here we extend the known distribution of the species toward the norther up Constitución, Maule Región include (locality 1; Table 1 and Figure 1) where it almost reaches the southern known locality of *A. longipilis* (see Valdez et al., 2020). *A. hirta* inhabits highly contrasting environmental conditions, including sclerophyllous forests, the humid temperate Valdivian and Magellan rainforest, and the arid Patagonian steppe. This wide geographic distribution includes areas that during the Quaternary were impacted, at varying degrees of intensity, by climatic fluctuations. Using a wide SNP panel derived from transcriptomes, we identified intraspecific lineages and described the genetic structure and demographic history of *A. hirta*. With this approach, we are contributing to undertake the transition promoted by Lessa et al. (2014) of studies on South American rodents toward the genomic era.

### Genetic Variation in *Abrothrix hirta* Across Its Geographic Distribution

The fact that *Abrothrix hirta* presents its genetic variation geographically structured was consistently depicted by three analytical approaches, namely, ML reconstruction, PCA, and Ancestry analysis. These lineages are the Maule, Araucarian, Valdivian, and Patagonian lineages (Figures 1C, 3, 4). The Araucarian lineage is represented by A single specimen (UACH 8180) from Malalcahuello (locality 3), whose distinction was shown in both the ML tree and the PCA (Figures 1C, 3). This specimen was not included in the Ancestry analysis because it requires multiple samples per each locality. Further sampling in pre-Andean areas of the Araucanía Region will help test the genetic distinction of this geographic area which is shown by specimen UACH 8180.

Other small mammals that are co-distributed with *Abrothrix hirta* also exhibit geographic structure. Albeit in a much smaller distribution area, the congeneric Valdivian forest endemic *A. manni* presents two main clades (Valdez and D’Elía, 2018). Another congeneric species, the olive soft-haired mouse *A. olivacea* presents three main clades in the study area, one in central and northern Chile, and the others in Patagonia and in Tierra del Fuego (Lessa et al., 2010). The southern pericote *Loxodontomys micropus* also presents segregated lineages in Patagonia (Cañón et al., 2010). Unlike these, several other small mammals within the geographic area of *Abrothrix hirta*, such as *Eligmodontia morgani*, *Reithrodon auritus*, and *Phyllotis xanthopygus* (Lessa et al., 2010), do not exhibit phylogeographic breaks. However, all these studies were based on unilocus mitochondrial genetic variation, with the consequent limitation of uncertainty about the possible distortion of demographic inferences due to the effect of natural selection. Our SNP-based approach allows us to confidently state that *A. hirta* presents distinct genetic lineages across its distribution.

Several phylogeographical breaks were detected in this study; however, given the gaps in our geographic sampling, we focused attention on two breaks located approximately in the same areas as other species also show phylogeographic breaks. The northernmost break separates the Maule lineage from the Araucarian, Valdivian and Patagonian lineages on the other (Figure 1). A finer-grain sampling is needed to precisely locate this break, but it roughly coincides with the general area of the break described for the small cat *Leopardus guigna* (Napolitano et al., 2014), the frog *Eupsophus roseus* (Correa et al., 2017), and the lizard *Liolaemus pictus* (Vera-Escalona et al., 2012). Since divergence times are not provided for all of the above studies, it is not possible to establish whether the divergences occurred at the same time, and therefore whether they were produced by the same event. At this latitude lie the rivers Itata and Bio Bio. For a small mammal such as *A. hirta*, the riverine barrier might have acted as a primary barrier, bisecting a previously continuous population, or as a secondary barrier, preventing the gene flow between already differenced populations (Patton and da Silva, 1998). In the case of *Abrothrix hirta*, considering that the rivers are most likely older than the divergence in the basal dichotomy (ca. 198 Mya; Figure 5) it is possible that a founder effect, likely from Central Chile southwards across these rivers would have promoted the differentiation between the Maule lineage and the Araucarian, Valdivian, and Patagonian lineages.

Another phylogeographic break of *Abrothrix hirta* is located at ca. 43°S, at Los Ríos Region in Chile and Río Negro and northern Chubut in Argentina (Figure 1). A break at this latitude was also observed in the olive soft-haired mouse *Abrothrix olivacea* (Lessa et al., 2010) and in the huemul *Hippocamelus bisulcus* (Marín et al., 2013a). The latitude 43° S is also an area of contact of two widely distributed lineages of the guanaco *Lama guanicoe* (Marín et al., 2013b). The frogs *Batrachyla leptopus* (Nuñez et al., 2020), *Pleurodema thaul* (Barria et al., 2020), and the lizard *Liolaemus petrophilus* (Fontanella et al., 2012) also present phylogeographic breaks in this area. Several of the species mentioned above, diverged into intraspecific lineages and subsequently showed demographic expansion of at least one of them toward Patagonia after the LGM. It is possible that the split depicted at this phylogeographical break would have originated by genetic divergence of populations isolated in at least two refugial areas during the Pleistocene glaciations in Central-southern Chile and Argentina, from where they expanded southwards. This hypothesis can be tested using denser spatial sampling and demographic modeling approaches.

Within the study area, several processes have been proposed as possible causes of the generation and/or maintenance of intraspecific lineages in terrestrial vertebrates, such as a riverine effect in species of mammals, reptiles, frogs, and plants (e.g., Torres-Pérez et al., 2007; Sallaberry-Pincheira et al., 2011; Vásquez et al., 2013; Palma et al., 2014; Viruel et al., 2014; Muñoz-Mendoza et al., 2017), habitat fragmentation prompted by climatic changes (e.g., Lessa et al., 2010; Valdez and D’Elía, 2018) and isolation-by-distance followed by population differentiation (Sersic et al., 2011). Based on the phylogeographic pattern described here, a combination of the mentioned processes is plausible to explain the genetic footprints of *Abrothrix hirta*. In particular, the fact that the genetic variation of *A. hirta* is nested within that of the Maule populations suggests a history of colonization from this area toward the south.

The main dichotomy within the Patagonian lineage is consistent with the scheme of northern-southern Patagonian clades noted in Lessa et al. (2010). Samples from Gan Gan (locality 7) comprise a northern lineage that extends northwards in Argentina up to Lago Quillén, Chubut, an area that is not covered by our sampling. Lessa’s souththern clade coincided with our Patagonian clade (excluding samples from locality 7). This general pattern of latitudinally replacing clades contrasts with the variation in *A. hirta* in terms of morphological variation that are associated with water economy. Naya et al. (2014) showed that populations from mesic Valdivian and Magellanic forests at the west slope of the Andes have larger body size and longer intestines relative to those from the xeric Patagonian steppe, east of the Andes. The same contrast between genetic structure and eco-morphological traits related to water economy was documented in the olive soft-haired mouse *Abrothrix olivacea* (Lessa et al., 2010; Giorello et al., 2018).

### Historical Demography of *Abrothrix hirta*

The range of *Abrothrix hirta* faced marked climatic changes during the Quaternary (Heusser, 1984; Rabassa et al., 1989). Glaciation cycles strongly impacted the species range, particularly in Patagonian. For instance, during the LGM the ice cap covered part of the Chilean territory, from sea level in southern Chiloé, northwards through the regions of Los Ríos and Los Lagos up to ca. 33°S (McCulloch et al., 2000). Coastal areas of Los Lagos, Los Ríos, and lowlands up to Bio Bio were ice-free, although areas that are currently forested had undergone a transition to subantarctic parklands and woodlands (Heusser et al., 1996; Moreno et al., 2001). In our analyses, the genetic footprints of *Abrothrix hirta* can be traced back in time up to ca. 210 Kya (Figure 5A), i.e., before the Middle Quaternary Glaciation (190–130 Kya). At that time, the ancestral population experienced an exponential growth before the divergence that originated the Maule lineage and the ancestor of the other lineages. During the Middle Quaternary Glaciation (190–130 Kya), the Early Late Quaternary Glaciation (98–75Kya), and the Middle Late Quaternary Glaciation (43–33 Kya) and the interglacial intervals among them, a progressive demographic reduction marked the history of the Maule lineage as well as that of the ancestor of the other lineages. Before and during the LGM population sizes persisted in markedly small populations in the Maule, Valdivian, and Patagonian lineages. Noteworthy is the fact that there has been gene flow during the last ca. 40 Ky, with strong levels roughly before and after the LGM, although genetic interchange between the Valdivian and Patagonian lineages also occurred during the last glaciation. Taking into consideration the geographic distribution of the Maule and Valdivian lineages in areas that were not impacted by late Pleistocene climate as in southern areas, it is expectable to register gene flow among those lineages. It is noteworthy that higher intensity of gene flow was inferred from the Valdivian lineage toward the Maule (see arrows in Figure 5). Regarding the Patagonian lineage, it is likely that the gene flow detected during the LGM would have taken place at the northern edge of its range. Again, finer-scale sampling would clarify these events. However, although geographically limited, the dataset used in this study provided information that single-locus studies cannot; for this reason, the scenario presented here represents a new generation of hypotheses about the demographic history of Patagonian rodents.

Since the LGM, populations in the northern part of the range (ca. 34° S–42°S) remained stable, while in the south (ca. 43°S–54°S), populations have expanded. The Maule and Valdivian lineages maintained their effective population size over the last ca. 15 Ky (Figure 5). In a neutral framework, a population that has remained stable through time accumulates more genetic variants, which increases its divergence due to random mutations. Concordant with this expectation are the results showing that the genetic diversity was higher at northern localities (Figure 2), and that branch lengths in the ML tree are longer in Maule and Valdivian lineages than those of the Patagonian lineage (Figure 1C), despite the geographic distribution of the latter is markedly larger than the distributions of Maule and Valdivian lineages (Figure 1A). Additionally, the genetic dispersions of the specimens that compose the Maule and Valdivian lineages are larger than that those shown by the specimens of the Patagonian lineage (Figure 3). Conversely, our results suggest a process of post-glaciation colonization of open and Magellanic forest areas of Patagonia, as population in these areas experienced a recent population increase (Figure 5), showed a pattern of isolation-by-distance), and are genetically less diverse (Figure 2; see also branch lengths in Figure 1C).

Putative Pleistocene refugial areas, from which populations expanded toward their current distribution range, have been identified in central-southern Chile. This is the case for small mammals, including the mice *Oligoryzomys longicaudatus* (Palma et al., 2012) and *Abrothrix manni* (Valdez and D’Elía, 2018), the amphibians *Batrachyla leptopus* (Nuñez et al., 2020) and *Rhinella arunco* (Vásquez et al., 2013); the reptiles *Liolaemys pictus* (Victoriano et al., 2008; Vera-Escalona et al., 2012) and *L. tenius*, *L. lemniscatus* (Victoriano et al., 2008) and the plants *Hypochaeris palustris* (Muellner et al., 2005) and *Nothofagus pumilio* (Mattera et al., 2020). Most of the studies reported demographic expansions after the LGM, indicating progressive population growth in the area over the past ca. 15,000 years. Moreover, besides the population increase, the pattern of colonization of Patagonian areas from northern refugial areas was inferred in other sigmodontine rodents, including *Abrothrix olivacea* (sensu Lessa et al., 2010; Cañón et al., 2014), *Eligmodontia morgani*, *Phyllotys xanthopygus*, and (Smith et al., 2001; Lessa et al., 2010). Although the latter also presents signs of population persistence in another refugium at Tierra del Fuego. Also, plant species exhibited similar historical patterns (e.g., *Hypochaeris incana* Tremetsberger et al., 2009; *Echinopsis chiloensis* Ossa et al., 2019). Taken as whole, three phylogeographic studies focused Patagonia revealed that the response of the biota to climatic oscillations is mostly species-specific. This fact justifies the efforts to unravel species genetic patterns not only to increase knowledge on local dynamics but also to make informed conservation decisions.

### Taxonomic Implications

The genetic patterns depicted in this study identified distinct lineages (Figure 1) among which there is gene flow (Figure 5); this is consistent with a taxonomical scheme where a single species is recognized. However, a scenario of multiples hybridizing species cannot be ruled out. Considering a single species framework, intraspecific lineages identified here might be recognized at the subspecies level. A study of the morphological and morphometric variation within and among the lineages described here will help determine the most appropriate taxonomical arrangement for these populations. Several taxa (e.g., *apta*, *moerens*, *suffusus*) are included under the synonymy of *A. hirta*. Our geographic coverage is limited and as such, it does not allow a proper association between available names and our identified lineages. Further studies on morphological and genetic variation are required to address this issue.

### Implications for Conservation

Phylogeographic and population genomic studies provide the way to uncover the geographic distribution of genetic diversity and the demographic history of target species. As early stated by Moritz (1994), when advancing the notion of Evolutionarily Significant Units, understanding the evolutionary history of the species, the number, composition, and distribution of intraspecific lineages are relevant for conservation purposes (see also Ryder, 1986; Supple and Shapiro, 2018). Each lineage, which besides taxonomic status can be considered as distinct ESU, has its own combination of genetic variants (including those that discriminate the genetic groups in Figures 1, 3, 4), therefore a distinct genetic pool, which in turn would allow different responses to future environmental changes. Additionally, it has been shown that the distinctive properties of intraspecific lineages affect the projections of species distribution models (i.e., the sum of the areas resulting from models built for each lineage separately is distinct to the area resulting when modeling the species as a whole) and therefore the accuracy of predictions in the context of the environmental change due to global warming and anthropogenic impact (Barria et al., 2020).

While the conservation status of *Abrothrix hirta* in Argentina is considered as of low concern (Teta and D’Elía, 2019), it has not been categorized by neither the International Union for Conservation of Nature (IUCN) nor the Chilean authorities via the Ministerio del Medio Ambiente. Populations of this species are still included under *Abrothrix longipilis*, following an old taxonomic scheme abandoned seven years ago (Teta and Pardiñas, 2014). This is problematic not only because it greatly overestimates the distribution of *A. longipilis*, but also because the true genetic diversity and distribution of the intraspecific lineages of *A. hirta* remain hidden from conservation concerns. Our results indicate that two of the four main lineages of *A. hirta* occur Chilean in protected areas. This is the case of the Araucarian lineage, which is sheltered by the national protected area Reserva Nacional Malalcahuello (locality 6; Figure 1A). It is also possible that individuals from this lineage are present in other nearby Chilean and Argentinean protected areas. The Valdivian lineage is also being protected; we collected individuals at Fundo San Martín, a small private conservation property of the Universidad Austral de Chile (locality 4; Figure 1A). Again, it is also possible that other protected areas harbor populations of this lineage in Chile and/or Argentina. We did not collect the Patagonian lineage at any protected area but given its large distribution and the several large protected areas existing in both Argentinean and Chilean Patagonia, we suggest that the status of this lineage to be of least concern. However, it is of concern to determine whether the Maule lineage is safeguarded. This is the most divergent lineage of *Abrothrix hirta* and the one with the highest genetic diversity (Figures 1, 2). We have not collected individuals from this lineage within any protected areas.

Central-southern Chile is listed as one of the 25 biodiversity hotspots for conservation priorities; it harbors a high level of endemism of species and genera (Myers et al., 2000; Arroyo et al., 2006). It is also true that central-southern Chile suffers high anthropogenic impact since the Spanish colonization five centuries ago (Armesto et al., 2001; Smith-Ramírez, 2004; Silva and Saavedra, 2018). Currently, intensive deforestation coupled with grazing by cattle, human-caused fires, and expansion of invasive species are some of the strongest threats for the local biodiversity (Armesto et al., 2001; Smith-Ramírez, 2004; Escobar et al., 2015). This situation rises the necessity of categorizing not only at the species level, but also at the intraspecific lineages, either in a context of recognized subspecies or not. This might be the case of several species, particularly those with wide geographic distribution, that are categorized as of least concern in southern South America. In this genomic era, with the astonishing technological advances at hand, it is possible to individualize intraspecific lineages, exposing their unique genetic legacy to avoid losing the richness of this heritage.

## Data Availability Statement

The data presented in the study are deposited in the EMBL-EBI European Variation Archive (EVA) repository, accession numbers PRJEB43642, ERZ1758066, ERZ1758067, and ERZ1758068; Bioproject PSUB014331; Biosamples SAMD00278635–SAMD00278678.

## Ethics Statement

The collection of some Chilean specimens was authorized by the Servicio Agrícola y Ganadero (permits 1231/2017, 5611/2013, 2164 and 5165); collection procedures were approved by the Universidad Austral de Chile (permits 04/11 and 296/2017). Other studied specimens were already housed in museum collections.

## Author Contributions

LV conceived and designed the experiments, performed the experiments, analyzed the data, prepared figures and tables, authored and reviewed drafts of the manscript, and approved the final draft. GD’E conceived and designed the experiments, authored and reviewed drafts of the manscript, and approved the final draft. Both authors contributed to the article and approved the submitted version.

## Conflict of Interest

The authors declare that the research was conducted in the absence of any commercial or financial relationships that could be construed as a potential conflict of interest. The reviewer BP declared a past co-authorship with one of the authors, GD’E, to the handling editor.

## Footnotes

1 http://www.bioinformatics.babraham.ac.uk/projects/trim_galore/

2 https://www.ncbi.nlm.nih.gov/nuccore/

3 https://omabrowser.org/oma/

4 http://samtools.github.io/bcftools/

5 https://github.com/isaacovercast/easySFS

## References

[B1] Achimón F. Johnson L. A. Cocucci A. A. Sérsic A. N. Baranzelli M. C. (2018). Species tree phylogeny, character evolution, and biogeography of the Patagonian genus *Anarthrophyllum Benth*. (*Fabaceae*). *Org. Divers. Evol.* 18 71–86. 10.1007/s13127-017-0355-1

[B2] Alexander D. H. Lange K. (2011). Enhancements to the ADMIXTURE algorithm for individual ancestry estimation. *BMC Bioinformatics* 12:246. 10.1186/1471-2105-12-246 21682921 PMC3146885

[B3] Armesto J. J. Rozzi R. Caspersen J. (2001). “Temperate forests of North and South America,” in *Global Biodiversity in a Changing Environment. Scenarios for the 21st Century*, eds Chapin F. S. Sala O. E. Huber-Sannwald E., (New York, NY: Springer).

[B4] Arroyo M. T. Marquet P. A. Marticorena C. Cavieres L. A. Squeo F. A. Simonetti J. (2006). “El hotspot chileno, prioridad mundial para la conservación. Diversidad de ecosistemas, ecosistemas terrestres,” in *Biodiversidad de Chile. Patrimonio y Desafíos. Ministerio del Medio Ambiente*, Santiago.

[B5] Avise J. C. (2009). Phylogeography: retrospect and prospect. *J. Biogeogr.* 36 3–15. 10.1111/j.1365-2699.2008.02032.x

[B6] Barria A. M. Zamorano D. Parada A. Labra F. A. Estay S. A. Bacigalupe L. D. (2020). The importance of intraspecific variation for niche differentiation and species distribution models: the ecologically diverse frog *Pleurodema thaul* as study case. *Evol. Biol.* 47 206–219. 10.1007/s11692-020-09510-0

[B7] Biersma E. M. Jackson J. A. Bracegirdle T. J. Griffiths H. Linse K. Convey P. (2018). Low genetic variation between South American and Antarctic populations of the bank-forming moss *Chorisodontium aciphyllum* (*Dicranaceae*). *Polar Biol.* 41 599–610. 10.1007/s00300-017-2221-1

[B8] Camacho C. Coulouris G. Avagyan V. Ma N. Papadopoulos J. Bealer K. (2009). BLAST+: architecture and applications. *BMC Bioinformatics* 10:421. 10.1186/1471-2105-10-421 20003500 PMC2803857

[B9] Cañón C. D’Elía G. Pardiñas U. F. Lessa E. P. (2010). Phylogeography of *Loxodontomys micropus* with comments on the alpha taxonomy of *Loxodontomys* (*Cricetidae*: *Sigmodontinae*). *J. Mammal.* 91 1449–1458. 10.1644/10-mamm-a-027.1

[B10] Cañón C. Mir D. Pardiñas U. F. J. Lessa E. P. D’Elía G. (2014). A multilocus perspective on the phylogenetic relationships and diversification of rodents of the tribe *Abrotrichini* (*Cricetidae*: *Sigmodontinae*). *Zool. Scr.* 43 443–454. 10.1111/zsc.12069

[B11] Canto J. Saldarria M. Yáñez J. (2017). Estudio craneométrico en *Abrothrix hirta* (Thomas, 1895) (*Rodentia*: *Cricetidae*): una aproximación desde la morfometría geométrica. *MNHN Chile* 66 101–123.

[B12] Casacci L. P. Barbero F. Balletto E. (2014). The “Evolutionarily Significant Unit” concept and its applicability in biological conservation. *Ital. J. Zool.* 81 182–193. 10.1080/11250003.2013.870240

[B13] Correa C. Vásquez D. Castro-Carrasco C. Zúñiga-Reinoso Á Ortiz J. C. Palma R. E. (2017). Species delimitation in frogs from South American temperate forests: the case of *Eupsophus*, a taxonomically complex genus with high phenotypic variation. *PLoS One* 12:e0181026. 10.1371/journal.pone.0181026 28809924 PMC5557580

[B14] Danecek P. McCarthy S. A. (2017). BCFtools/csq: haplotype-aware variant consequences. *Bioinformatics* 33 2037–2039. 10.1093/bioinformatics/btx100 28205675 PMC5870570

[B15] D’Elía G. Pardiñas U. F. Jayat J. P. Salazar-Bravo J. (2008). Systematics of *Necromys* (Rodentia, Cricetidae, Sigmodontinae): species limits and groups, with comments on historical biogeography. *J. Mammal.* 89, 778–790.

[B16] Dobin A. Davis C. A. Schlesinger F. Drenkow J. Zaleski C. Jha S. (2013). STAR: ultrafast universal RNA-seq aligner. *Bioinformatics* 29 15–21. 10.1093/bioinformatics/bts635 23104886 PMC3530905

[B17] Escobar M. A. Uribe S. V. Chiappe R. Estades C. F. (2015). Effect of clearcutting operations on the survival rate of a small mammal. *PLoS One* 10:e0118883. 10.1371/journal.pone.0118883 25748217 PMC4352083

[B18] Femenias M. M. Avila L. J. Sites J. W. Jr. Morando M. (2020). The enigmatic Leiosaurae clade: phylogeography, species delimitation, phylogeny and historical biogeography of its southernmost species. *Mol. Phylogenet. Evol.* 144:106725. 10.1016/j.ympev.2019.106725 31884086

[B19] Fitzpatrick M. J. Edelsparre A. H. (2018). The genomics of climate change. *Science* 359 29–30. 29301999 10.1126/science.aar3920

[B20] Fontanella F. M. Feltrin N. Avila L. J. Sites J. W. Jr. Morando M. (2012). Early stages of divergence: phylogeography, climate modeling, and morphological differentiation in the South American lizard *Liolaemus petrophilus* (*Squamata*: *Liolaemidae*). *Ecol. Evol.* 2 792–808. 10.1002/ece3.78 22837827 PMC3399201

[B21] Frichot E. François O. (2015). LEA: an R package for landscape and ecological association studies. *MEE* 6 925–929. 10.1111/2041-210x.12382

[B22] Gaston K. J. Spicer J. I. (2004). *Biodiversity: An Introduction*, 2nd Edn. Oxford: Blackwell Publishing.

[B23] Giorello F. M. Feijoo M. D’Elía G. Naya D. E. Valdez L. Opazo J. C. (2018). An association between differential expression and genetic divergence in the Patagonian olive mouse (*Abrothrix olivacea*). *Mol. Ecol.* 27 3274–3286. 10.1111/mec.14778 29940092

[B24] Giorello F. M. Feijoo M. D’Elía G. Valdez L. Opazo J. C. Varas V. (2014). Characterization of the kidney transcriptome of the South American olive mouse *Abrothrix olivacea*. *BMC Genomics* 15:446. 10.1186/1471-2164-15-446 24909751 PMC4189146

[B25] Grabherr M. G. Haas B. J. Yassour M. Levin J. Z. Thompson D. A. Amit I. (2011). Full-length transcriptome assembly from RNA-Seq data without a reference genome. *Nat. Biotechnol.* 29 644–652. 10.1038/nbt.1883 21572440 PMC3571712

[B26] Gutenkunst R. N. Hernandez R. D. Williamson S. H. Bustamante C. D. (2009). Inferring the joint demographic history of multiple populations from multidimensional SNP frequency data. *PLoS Genet.* 5:e1000695. 10.1371/journal.pgen.1000695 19851460 PMC2760211

[B27] Heusser C. J. (1984). Late-glacial-holocene climate of the lake district of chile. *Quat. Res.* 22 77–90. 10.1016/0033-5894(84)90008-5

[B28] Heusser C. J. Lowell T. V. Heusser L. E. Hauser A. Andersen B. G. Denton G. H. (1996). Full−glacial—late−glacial palaeoclimate of the Southern Andes: evidence from pollen, beetle and glacial records. *J. Quat. Sci.* 11 173–184. 10.1002/(sici)1099-1417(199605/06)11:3<173::aid-jqs237>3.0.co;2-5

[B29] Hoang D. T. Chernomor O. Von Haeseler A. Minh B. Q. Vinh L. S. (2018). UFBoot2: improving the ultrafast bootstrap approximation. *Mol. Biol. Evol.* 35 518–522. 10.1093/molbev/msx281 29077904 PMC5850222

[B30] Jouganous J. Long W. Ragsdale A. P. Gravel S. (2017). Inferring the joint demographic history of multiple populations: beyond the diffusion approximation. *Genetics* 206 1549–1567. 10.1534/genetics.117.200493 28495960 PMC5500150

[B31] Kelt D. A. Meserve P. L. Lang B. K. (1994). Quantitative habitat associations of small mammals in a temperate rainforest in southern Chile: empirical patterns and the importance of ecological scale. *J. Mammal.* 75 890–904. 10.2307/1382471

[B32] Langmead B. Salzberg S. L. (2012). Fast gapped-read alignment with Bowtie 2. *Nat. Methods* 9 357–379. 10.1038/nmeth.1923 22388286 PMC3322381

[B33] Lessa E. P. Cook J. A. D’Elía G. Opazo J. C. (2014). Rodent diversity in South America: transitioning into the genomics era. *Front. Ecol. Evol.* 2:39. 10.3389/fevo.2014.00039

[B34] Lessa E. P. D’Elía G. Pardinas U. F. (2010). Genetic footprints of late Quaternary climate change in the diversity of Patagonian-Fueguian rodents. *Mol. Ecol.* 19 3031–3037. 10.1111/j.1365-294x.2010.04734.x 20618900

[B35] Marín J. C. Varas V. Vila A. R. López R. Orozco-terWengel P. Corti P. (2013a). Refugia in Patagonian fjords and the eastern Andes during the Last Glacial Maximum revealed by huemul (*Hippocamelus bisulcus*) phylogeographical patterns and genetic diversity. *J. Biogeogr.* 40 2285–2298.

[B36] Marín J. C. González B. A. Poulin E. Casey C. S. Johnson W. E. (2013b). The influence of the arid Andean high plateau on the phylogeography and population genetics of guanaco (*Lama guanicoe*) in South America. *Mol. Ecol.* 22 463–482. 10.1111/mec.12111 23206254 PMC3549358

[B37] Mattera M. G. Pastorino M. J. Lantschner M. V. Marchelli P. Soliani C. (2020). Genetic diversity and population structure in *Nothofagus pumilio*, a foundation species of Patagonian forests: defining priority conservation areas and management. *Sci. Rep.* 10 1–13. 33159157 10.1038/s41598-020-76096-0 PMC7648826

[B38] McCulloch R. D. Bentley M. J. Purves R. S. Hulton N. R. Sugden D. E. Clapperton C. M. (2000). Climatic inferences from glacial and palaeoecological evidence at the last glacial termination, southern South America. *J. Quat. Sci.* 15 409–417. 10.1002/1099-1417(200005)15:4<409::aid-jqs539>3.0.co;2-#

[B39] McKenna A. Hanna M. Banks E. Sivachenko A. Cibulskis K. Kernytsky A. (2010). The genome analysis toolkit: a MapReduce framework for analyzing next-generation DNA sequencing data. *Genome Res.* 20 1297–1303. 10.1101/gr.107524.110 20644199 PMC2928508

[B40] Moreno P. I. Jacobson G. L. Lowell T. V. Denton G. H. (2001). Interhemispheric climate links revealed by a late-glacial cooling episode in southern Chile. *Nature* 409 804–808. 10.1038/35057252 11236990

[B41] Moritz C. (1994). Defining ‘evolutionarily significant units’ for conservation. *Trends Ecol. Evol.* 9 373–375. 10.1016/0169-5347(94)90057-4 21236896

[B42] Moritz C. (2002). Strategies to protect biological diversity and the evolutionary processes that sustain it. *Syst. Biol.* 51 238–254. 10.1080/10635150252899752 12028731

[B43] Muellner A. N. Tremetsberger K. Stuessy T. Baeza C. M. (2005). Pleistocene refugia and recolonization routes in the southern Andes: insights from *Hypochaeris palustris* (*Asteraceae*. *Lactuceae*). *Mol. Ecol.* 14 203–212. 10.1111/j.1365-294x.2004.02386.x 15643964

[B44] Muñoz-Mendoza C. D’Elía G. Panzera A. Villalobos-Leiva A. Sites J. W. Jr. Victoriano P. F. (2017). Geography and past climate changes have shaped the evolution of a widespread lizard from the Chilean hotspot. *Mol. Phylogenet. Evol.* 116 157–171. 10.1016/j.ympev.2017.08.016 28887150

[B45] Myers N. Mittermeier R. A. Mittermeier C. G. Da Fonseca G. A. Kent J. (2000). Biodiversity hotspots for conservation priorities. *Nature* 403 853–858. 10.1038/35002501 10706275

[B46] Napolitano C. Johnson W. E. Sanderson J. O’Brien S. J. Hoelzel A. R. Freer R. (2014). Phylogeography and population history of *Leopardus guigna*, the smallest American felid. *Conserv. Genet.* 15 631–653. 10.1007/s10592-014-0566-3

[B47] Naya D. E. Feijoo M. Lessa E. P. Pardiñas U. F. Teta P. Tomasco I. H. (2014). Digestive morphology of two species of *Abrothrix* (*Rodentia*, *Cricetidae*): comparison of populations from contrasting environments. *J. Mammal.* 95 1222–1229. 10.1644/13-mamm-a-261

[B48] Nguyen L. T. Schmidt H. A. Von Haeseler A. Minh B. Q. (2015). IQ-TREE: a fast and effective stochastic algorithm for estimating maximum-likelihood phylogenies. *Mol. Biol. Evol.* 32 268–274. 10.1093/molbev/msu300 25371430 PMC4271533

[B49] Nicola M. V. Johnson L. A. Pozner R. (2019). Unraveling patterns and processes of diversification in the South Andean-Patagonian Nassauvia subgenus *Strongyloma* (*Asteraceae*, *Nassauvieae*). *Mol. Phylogenet. Evol.* 136 164–182. 10.1016/j.ympev.2019.03.004 30858079

[B50] Norambuena H. V. Van Els P. Muñoz-Ramírez C. P. Victoriano P. F. (2018). First steps towards assessing the evolutionary history and phylogeography of a widely distributed Neotropical grassland bird (*Motacillidae*: *Anthus correndera*). *PeerJ* 6:e5886. 10.7717/peerj.5886 30498628 PMC6252069

[B51] Noskova E. Ulyantsev V. Koepfli K. P. O’Brien S. J. Dobrynin P. (2020). GADMA: Genetic algorithm for inferring demographic history of multiple populations from allele frequency spectrum data. *GigaScience* 9:giaa005. 10.1093/gigascience/giaa005 PMC7049072 32112099

[B52] Nuñez J. J. Suárez-Villota E. Y. Quercia C. A. Olivares A. P. Sites J. W. Jr. (2020). Phylogeographic analysis and species distribution modelling of the wood frog *Batrachyla leptopus* (*Batrachylidae*) reveal interglacial diversification in south western Patagonia. *PeerJ* 8:e9980. 10.7717/peerj.9980 33083116 PMC7546244

[B53] Nuñez J. J. Wood N. K. Rabanal F. E. Fontanella F. M. Sites J. W. Jr. (2011). Amphibian phylogeography in the Antipodes: refugia and postglacial colonization explain mitochondrial haplotype distribution in the Patagonian frog *Eupsophus calcaratus* (Cycloramphidae). *Mol. Phylogenet. Evol.* 58, 343–352. 10.1016/j.ympev.2010.11.026 21145400

[B54] Ossa C. G. Montenegro P. Larridon I. Pérez F. (2019). Response of xerophytic plants to glacial cycles in southern South America. *Ann. Bot.* 124 15–26. 10.1093/aob/mcy235 30715148 PMC6676391

[B55] Palma R. E. Boric-Bargetto D. Torres-Perez F. Hernandez C. E. Yates T. L. (2012). Glaciation effects on the phylogeographic structure of *Oligoryzomys longicaudatus* (*Rodentia*: *Sigmodontinae*) in the Southern Andes. *PLoS One* 7:e32206. 10.1371/journal.pone.0032206 22396751 PMC3291571

[B56] Palma R. E. Boric−Bargetto D. Jayat J. P. Flores D. A. Zeballos H. Pacheco V. (2014). Molecular phylogenetics of mouse opossums: new findings on the phylogeny of *Thylamys* (*Didelphimorphia*, *Didelphidae*). *Zool. Scr.* 43 217–234.

[B57] Palma R. E. Cancino R. A. Rodríguez-Serrano E. (2010). Molecular systematics of *Abrothrix longipilis* (Rodentia: Cricetidae: Sigmodontinae) in Chile. *J. Mammal.* 91, 1102–1111. 10.1644/10-MAMM-A-031.1

[B58] Paradis E. (2010). pegas: an R package for population genetics with an integrated–modular approach. *Bioinformatics* 26 419–420. 10.1093/bioinformatics/btp696 20080509

[B59] Patterson B. D. Meserve P. L. Lang B. K. (1990). Quantitative habitat associations of small mammals along an elevational transect in temperate rainforests of Chile. *J. Mammal.* 71 620–633. 10.2307/1381803

[B60] Patterson B. D. Teta P. Smith M. F. (2015). “Genus *Abrothrix* Waterhouse, 1837,” in *Mammals of South America*, eds Patton J. L. Pardiñas U. F. J. D’Elía G., (Chicago, IL: The University of Chicago Press), 2.

[B61] Patton J. L. da Silva M. N. F. (1998). “Rivers, refuges, and ridges: the geography of speciation in Amazonian mammals,” in *Endless Forms: Species and Speciation*, eds Howard D. Berlocher S., (New York, NY: Oxford University Press).

[B62] Pebesma E. Bivand R. S. (2005). S classes and methods for spatial data: the sp package. *R News* 5 9–13.

[B63] Pfeifer B. Wittelsbuerger U. Ramos-Onsins S. E. Lercher M. J. (2014). PopGenome: an efficient swiss army knife for population genomic analyses in R. *Mol. Biol. Evol.* 31 1929–1936. 10.1093/molbev/msu136 24739305 PMC4069620

[B64] Premoli A. C. Mathiasen P. Kitzberger T. (2010). Southern-most *Nothofagus* trees enduring ice ages: genetic evidence and ecological niche retrodiction reveal high latitude (54 S) glacial refugia. *Palaeogeogr. Palaeoclimatol. Palaeoecol.* 298 247–256. 10.1016/j.palaeo.2010.09.030

[B65] Purcell S. Neale B. Todd-Brown K. Thomas L. Ferreira M. A. Bender D. (2007). PLINK: a tool set for whole-genome association and population-based linkage analyses. *Am. J. Hum. Genet.* 81 559–575. 10.1086/519795 17701901 PMC1950838

[B66] Rabassa J. Heusser C. Rutter N. (1989). Late-glacial and holocene of argentine tierra del fuego. *Q. South Am. Antar. Peninsula* 7 327–351. 10.1201/9781003079361-17

[B67] Ruzzante D. E. Simons A. P. McCracken G. R. Habit E. Walde S. J. (2020). Multiple drainage reversal episodes and glacial refugia in a Patagonian fish revealed by sequenced microsatellites. *Proc. Biol. Sci.* 287:20200468. 10.1098/rspb.2020.0468 PMC7341911 32486985

[B68] Ruzzante D. E. Walde S. J. Cussac V. E. Dalebout M. L. Seibert J. Ortubay S. (2006). Phylogeography of the Percichthyidae (Pisces) in Patagonia: roles of orogeny, glaciation, and volcanism. *Mol. Ecol.* 15, 2949–2968. 10.1111/j.1365-294X.2006.03010.x 16911213

[B69] Ryder O. A. (1986). Species conservation and systematics: the dilemma of the subspecies. *Trends Ecol. Evol.* 1 9–10. 10.1016/0169-5347(86)90059-5

[B70] Sallaberry-Pincheira N. Garin C. F. González-Acuña D. Sallaberry M. A. Vianna J. A. (2011). Genetic divergence of Chilean long−tailed snake (*Philodryas chamissonis*) across latitudes: conservation threats for different lineages. *Divers. Distrib.* 17 152–162. 10.1111/j.1472-4642.2010.00729.x

[B71] Sersic A. N. Cosacov A. Cocucci A. A. Johnson L. A. Pozner R. Avila L. J. (2011). Emerging phylogeographical patterns of plants and terrestrial vertebrates from Patagonia. *Biol. J. Linn.* 103 475–494. 10.1111/j.1095-8312.2011.01656.x

[B72] Sikes R. S. Animal Care and Use Committee of the American Society of Mammalogists, (2016). 2016 Guidelines of the American Society of Mammalogists for the use of wild mammals in research and education. *J. Mammal* 97 663–688. 10.1093/jmammal/gyw078 29692469 PMC5909806

[B73] Silva C. Saavedra B. (2018). “El hombre y la Biodiversidad. 6.1. Actividades productivas y biodiversidad,” in *Biodiversidad de Chile. Patrimonio y desafíos. Ministerio del Medio Ambiente*, Santiago.

[B74] Smith M. F. Kelt D. A. Patton J. L. (2001). Testing models of diversification in mice in the *Abrothrix olivaceus*/*xanthorhinus* complex in Chile and Argentina. *Mol. Ecol.* 10 397–405. 10.1046/j.1365-294x.2001.01183.x 11298954

[B75] Smith-Ramírez C. (2004). The Chilean coastal range: a vanishing center of biodiversity and endemism in South American temperate rainforests. *Biodivers. Conserv.* 13 373–393. 10.1023/b:bioc.0000006505.67560.9f

[B76] Supple M. A. Shapiro B. (2018). Conservation of biodiversity in the genomics era. *Genome Biol.* 19 1–12. 30205843 10.1186/s13059-018-1520-3 PMC6131752

[B77] Teta P. Cañón C. Patterson B. D. Pardiñas U. F. (2017). Phylogeny of the tribe Abrotrichini (Cricetidae, Sigmodontinae): integrating morphological and molecular evidence into a new classification. *Cladistics* 33, 153–182. 10.1111/cla.12164 34710969

[B78] Teta P. D’Elía G. (2019). “Abrothrix hirta,” in *Categorización 2019 de los Mamíferos de Argentina Según su Riesgo de Extinción. Lista Roja de los Mamíferos de Argentina*, ed SAyDS–SAREM. Avaliable at: http://cma.sarem.org.ar. (accessed November 11, 2020).

[B79] Teta P. Pardiñas U. F. (2014). Variación morfológica cualitativa y cuantitativa en *Abrothrix longipilis* (*Cricetidae*, *Sigmodontinae*). *Mastozool. Neotrop.* 21 291–309.

[B80] Torres-Pérez F. Lamborot M. Boric-Bargetto D. Hernández C. E. Ortiz J. C. Palma R. E. (2007). Phylogeography of a mountain lizard species: an ancient fragmentation process mediated by riverine barriers in the *Liolaemus monticola* complex (*Sauria*: *Liolaemidae*). *J. Zool. Syst. Evol. Res* 45 72–81. 10.1111/j.1439-0469.2006.00392.x

[B81] Tremetsberger K. Urtubey E. Terrab A. Baeza C. M. Ortiz M. A. Talavera M. (2009). Pleistocene refugia and polytopic replacement of diploids by tetraploids in the Patagonian and Subantarctic plant *Hypochaeris incana* (*Asteraceae*. *Cichorieae*). *Mol. Ecol.* 18 3668–3682. 10.1111/j.1365-294x.2009.04298.x 19674310

[B82] Valdez L. D’Elía G. (2018). Local persistence of Mann’s soft-haired mouse *Abrothrix manni* (*Rodentia*, *Sigmodontinae*) during Quaternary glaciations in southern Chile. *PeerJ* 6:e6130. 10.7717/peerj.6130 30588409 PMC6302793

[B83] Valdez L. Giorello F. Feijoo M. Opazo J. C. Lessa E. P. Naya D. E. (2015). Characterization of the kidney transcriptome of the long-haired mouse *Abrothrix hirta* (*Rodentia*, *Sigmodontinae*) and comparison with that of the olive mouse *A. olivacea*. *PLoS One* 10:e0121148. 10.1371/journal.pone.0121148 25860131 PMC4393222

[B84] Valdez L. Quiroga-Carmona M. D’Elía G. (2020). Genetic variation of the Chilean endemic long-haired mouse Abrothrix longipilis (*Rodentia*, *Supramyomorpha*, *Cricetidae*) in a geographical and environmental context. *PeerJ* 8:e9517. 10.7717/peerj.9517 32742796 PMC7369023

[B85] Vásquez D. Correa C. Pastenes L. Palma R. E. Méndez M. A. (2013). Low phylogeographic structure of *Rhinella arunco* (*Anura*: *Bufonidae*), an endemic amphibian from the Chilean Mediterranean hotspot. *Zool. Stud.* 52:35. 10.1186/1810-522x-52-35

[B86] Vera-Escalona I. D’Elía G. Gouin N. Fontanella F. M. Muñoz-Mendoza C. Sites J. W. Jr. (2012). Lizards on ice: evidence for multiple refugia in *Liolaemus pictus* (*Liolaemidae*) during the Last Glacial Maximum in the Southern Andean Beech Forests. *PLoS one* 7:e48358. 10.1371/journal.pone.0048358 23209552 PMC3507886

[B87] Victoriano P. F. Ortiz J. C. Benavides E. Adams B. J. Sites J. W. Jr. (2008). Comparative phylogeography of codistributed species of Chilean *Liolaemus* (*Squamata*: *Tropiduridae*) from the central−southern Andean range. *Mol. Ecol.* 17 2397–2416. 10.1111/j.1365-294x.2008.03741.x 18430148

[B88] Viruel J. Catalan P. Segarra-Moragues J. G. (2014). Latitudinal environmental niches and riverine barriers shaped the phylogeography of the central Chilean endemic *Dioscorea humilis* (*Dioscoreaceae*). *PLoS One* 9:e110029. 10.1371/journal.pone.0110029 25295517 PMC4190404

[B89] Wouters M. Huybrechts T. Huys R. De Rore S. Sanders S. Umans E. (2002). “PICARD: platform concepts for prototyping and demonstration of high speed communication systems,” in *Proceedings of the 13th IEEE International Workshop on Rapid System Prototyping (RSP’02)*, Darmstadt, 166–170.

[B90] Zemlak T. S. Habit E. M. Walde S. J. Battini M. A. Adams E. D. Ruzzante D. E. (2008). Across the southern Andes on fin: glacial refugia, drainage reversals and a secondary contact zone revealed by the phylogeographical signal of *Galaxias platei* in Patagonia. *Mol. Ecol.* 17 5049–5061. 10.1111/j.1365-294x.2008.03987.x 19017262

