# Supplementary figures and images for: Genetic Diversity and Demographic History of the Shaggy Soft-Haired Mouse Abrothrix hirta (Cricetidae; Abrotrichini)
Source: Front Genet. 2021 Mar 24;12:642504. doi: 10.3389/fgene.2021.642504 (PMC8024643; doi:10.3389/fgene.2021.642504)

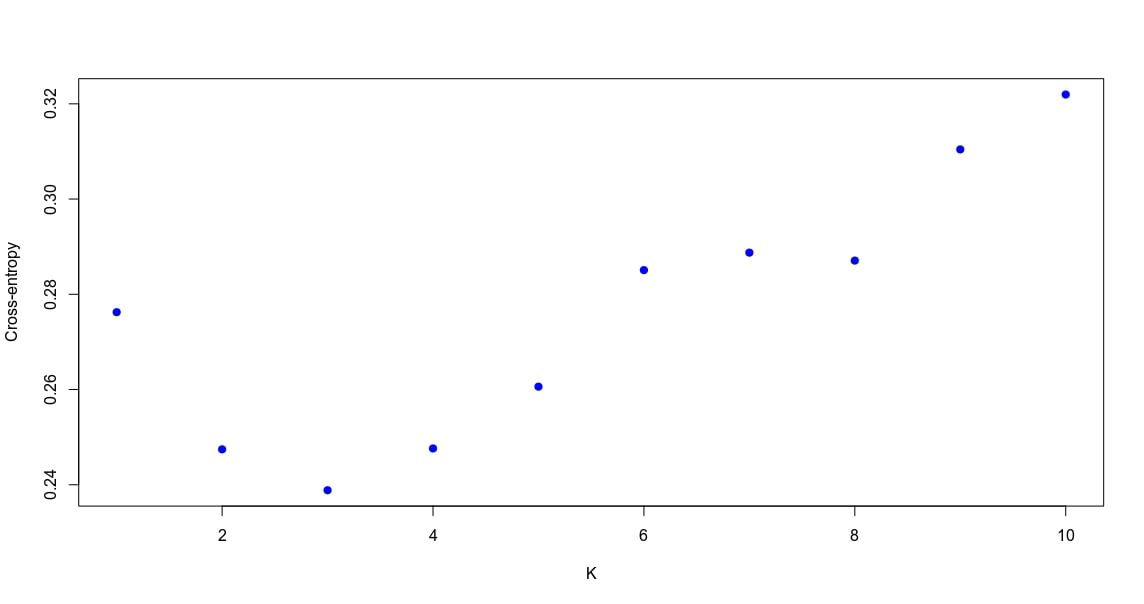

Supplement: Supplementary file 2 [file Image_1.JPEG]
